# Expression of *NDRG2 *is down-regulated in high-risk adenomas and colorectal carcinoma

**DOI:** 10.1186/1471-2407-7-192

**Published:** 2007-10-12

**Authors:** Anders Lorentzen, Lotte K Vogel, Rikke H Lewinsky, Mona Sæbø, Camilla F Skjelbred, Sine Godiksen, Geir Hoff, Kjell M Tveit, Inger Marie Bowitz Lothe, Tone Ikdahl, Elin H Kure, Cathy Mitchelmore

**Affiliations:** 1 Eucaryotic Cell Biology Research Group, Department of Science, Roskilde University, Roskilde, Denmark; 2 Department of Cellular and Molecular Medicine, University of Copenhagen, Blegdamsvej 3, Denmark; 3 Telemark University College, Faculty of Arts and Sciences, Bø i Telemark, Norway; 4 Department of Laboratory Medicine, Section of Medical Genetics, Telemark Hospital, Skien, Norway; 5 Department of Medicine, Telemark Hospital, Skien, Norway; 6 Department of Pathology, Ullevaal University Hospital, 0407 Oslo, Norway; 7 The Cancer Center, Ullevaal University Hospital, 0407 Oslo, Norway

## Abstract

**Background:**

It has recently been shown that *NDRG2 *mRNA is down-regulated or undetectable in several human cancers and cancer cell-lines. Although the function of NDRG2 is unknown, high *NDRG2 *expression correlates with improved prognosis in high-grade gliomas. The aim of this study has been to examine *NDRG2 *mRNA expression in colon cancer. By examining affected and normal tissue from individuals with colorectal adenomas and carcinomas, as well as in healthy individuals, we aim to determine whether and at which stages *NDRG2 *down-regulation occurs during colonic carcinogenesis.

**Methods:**

Using quantitative RT-PCR, we have determined the mRNA levels for *NDRG2 *in low-risk (n = 15) and high-risk adenomas (n = 57), colorectal carcinomas (n = 50) and corresponding normal tissue, as well as control tissue from healthy individuals (n = 15). *NDRG2 *levels were normalised to *β-actin*.

**Results:**

*NDRG2 *mRNA levels were lower in colorectal carcinomas compared to normal tissue from the control group (p < 0.001). When comparing adenomas/carcinomas with adjacent normal tissue from the same individual, *NDRG2 *expression levels were significantly reduced in both high-risk adenoma (p < 0.001) and in colorectal carcinoma (p < 0.001). There was a trend for *NDRG2 *levels to decrease with increasing Dukes' stage (p < 0.05).

**Conclusion:**

Our results demonstrate that expression of *NDRG2 *is down-regulated at a late stage during colorectal carcinogensis. Future studies are needed to address whether *NDRG2 *down-regulation is a cause or consequence of the progression of colorectal adenomas to carcinoma.

## Background

N-myc Downstream Regulated Gene 2 (*NDRG2*) is a member of a recently identified gene family which has been implicated in human nervous system disorders and cancer [1]. Although the four members of this family contain a putative α/β-hydrolase fold, it is unclear whether or not they have enzymatic activity [2]. *NDRG1 *was first identified as a gene under negative regulation by N-myc in early mouse development [3]. *NDRG2 *was identified through sequence homology and is implicated in cell growth, differentiation and neurodegeneration [4-7]. Recently, it has been shown that expression of *NDRG2 *is transcriptionally repressed by c-Myc [8].

Several studies have suggested that *NDRG2 *mRNA is down-regulated or undetectable in a number of human cancers and cancer cell-lines [4,9-11]. Semiquantitative RT-PCR was used to demonstrate that *NDRG2 *expression levels were reduced in squamous cell carcinoma, pancreatic cancer and glioblastoma compared to normal tissue [4,9,11]. *NDRG2 *expression levels in gliomas and meningiomas were significantly attenuated in high-grade compared to low-grade tumors [4,10]. In meningiomas, higher expression of *NDRG2 *mRNA correlated with clinically less aggressive tumors [10]. Furthermore, *NDRG2 *was identified as a gene whose expression in high-grade gliomas was positively correlated with survival [12]. Forced *NDRG2 *overexpression in a human glioblastoma cell-line markedly inhibited cell proliferation [4]. These findings implicate *NDRG2 *as a possible tumor suppressor gene.

Prompted by the finding that *NDRG2 *expression correlates inversely with tumor grade in various cancers, we set out to analyse *NDRG2 *mRNA expression during colorectal carcinogenesis in humans.

## Methods

### Subject population

The KAM cohort (Kolorektal cancer, Arv og Miljø) is based primarily on the screening group of the Norwegian Colorectal Cancer Prevention study (the NORCCAP study, ID number at Clinicaltrials.gov NCT00119912) in the county of Telemark, Norway [13,14]. Additionally, a series of colorectal cancer cases were recruited to the KAM cohort from routine clinical work at Telemark Hospital and Ulleval University Hospital in Oslo. A total of 20,780 men and women, age distribution 50–64 years, randomly drawn from the population registries in Oslo (urban) and the county of Telemark (mixed urban and rural) were invited to have a flexible sigmoidoscopy (FS) screening examination with or without (1:1) an additional faecal occult blood test (FOBT). A total of 777 (4%) individuals were excluded according to the exclusion criteria [13]. The KAM biobank currently consists of 234 colorectal cancer, 1044 adenoma (229 high-risk, 762 low-risk and 53 hyperplastic polyps) and 400 control specimens. Controls were defined as individuals with normal findings at FS. The KAM study is approved by the Regional Committee for Medical Research Ethics and the Norwegian Data Inspectorate. In the present study we have analyzed carcinomas (n = 50), adenomas (n = 72) and controls (n = 15). Each case was classified according to the degree of malignancy. A sample of control tissue was collected 30 cm from the anus of patients with adenomas, whereas two samples of control tissue were taken from the surgical specimen from patients with carcinomas: one sample in close proximity (normal adjacent) and one sample as far away from the tumor as possible (normal distant). Control samples were collected from individuals without adenomas or carcinomas. The histology of the adenomas was examined by two histopathologists independently. The degree of dysplasia was determined as either mild/moderate (n = 52) or severe (n = 20). The two pathologists reached the same conclusion in all cases. Furthermore, adenomas were classified as either low-risk (n = 15) or high-risk (n = 57). A high-risk adenoma is defined as an adenoma measuring ≥ 10 mm in diameter and/or with villous components and/or showing severe dysplasia [13]. The vast majority of CRC samples had 75–80% tumor cells surrounded by stroma, as evaluated by hematoxylin and eosin staining by a pathologist. The distribution of gender and age among controls and cases with colonic carcinoma or adenoma are shown in Table 1.

**Table 1:** Characteristics of cases and healthy persons in this study.

	**Controls**	**Cases**		
		**Low-risk adenomas**	**High-risk adenomas^1^**	**Carcinomas**

	**n = 15**	**n = 15**	**n = 57**	**n = 50**
**Men**	**5**	**12**	**40**	**31**
**Women**	**10**	**3**	**17**	**19**
**Mean age**^2 ^(SD)	**57.3 **(4.9)	**56.7 **(4.4)	**56.4 **(3.8)	**71.8 **(10.5)

^1^A high-risk adenoma is defined as an adenoma measuring ≥ 10 mm in diameter and/or with villous components and/or showing severe dysplasia.

^2^There are significant differences in age among the four groups of healthy and affected individuals at 95% confidence level (Kruskal-Wallis test).

### Cancer Profiling Array (CPA)

The Cancer Profiling Array II (Clontech) was hybridised with 50 ng of radioactively labelled *NDRG2 *probe according to the manufacturer's instructions. The 460 bp *NDRG2 *probe was generated by PCR using the primers 5'CTCACTCTGTGGAGACACCAT3' and 5'GGGTGATATCACCTCCACGCT3'. The hybridised array was exposed to a phosphorimaging screen for 24 hours and the intensity of each spot was quantified using ImageQuant (Molecular Dynamics). The CPA consists of paired cDNA samples generated from the total RNA of normal and tumor tissue. Because the array is normalised for several housekeeping genes, quantification of the hybridisation signal provides an estimate of relative transcript abundance.

### RT-PCR

Total RNA was purified from tissue as recommended by the manufacturers using an e.z.n.a. Gel Extraction kit (Omega Biotek). The tissue had been snap-frozen in liquid N_2 _and stored at -80°C before RNA purification. RNA purification included a DNAse treatment. The cDNA synthesis was performed on approximately 200 ng RNA per 10 μl using the High Capacity cDNA Reverse Transcription kit (Applied Biosystems, Nærum, Denmark). Quantitative RT-PCR was performed on an ABI7500 sequence detection system (Applied Biosystems) in Universal Mastermix (Applied Biosystems) using 220 nM probe and 700 nM primers for *NDRG2*. *NDRG2 *primers were NDRG2F: 5'CGATCCTTACCTACCACGATGTG3' and NDRG2R: 5'GCATGTCCTCGAACTGAAACAGT3' and the probe was 5'FAM-CTCAACTATAAATCTTGCTTCC-MGB-NFQ-3'. Primers were designed using Primer Express v3.0 Software and obtained from DNA Technology A/S. Primers were designed within different exons and with a probe covering the exon-exon border to prevent amplification of genomic DNA. The probe recognises all splice forms of NDRG2. *β-actin *primers and probe were obtained from Applied Biosystems. In a validation experiment using a control sample, a dilution series was produced and assayed for *NDRG2 *and *β-actin *expression as described in the comparative C_t _method [15]. When C_t _values were plotted against log dilution it was shown that the assays are quantitative over a range of 128-fold dilution for both *NDRG2 *and *β-actin *and that the PCR reactions have similar efficiencies provided that a threshold of 0.2 is used for *β-actin*, while the threshold was 0.07 for *NDRG2*. The threshold is a fixed fluorescence signal above the baseline. The C_t _value of a sample is determined as the fractional cycle number when the sample's fluorescence signal exceeds the threshold. The threshold is thus assay-specific, determined in the validation experiment and depends on the background of the individual assay.

*NDRG2 *and *β-actin *mRNAs were quantified separately in triplicates. The average standard deviations on triplicates were 15% and 11% for *NDRG2 *and *β-actin *respectively. The standard deviation on repeated measurements of the same sample (internal control) in separate experiments was 16% for *NDRG2*, indicating the day-to-day variation of the assay. Negative controls (where the RNA was not converted into cDNA) and positive controls were included in all sets. Two independent PCR reactions of 28 samples (5%) yielded a correlation coefficient of 0.95, indicating a high reproducibility of the assay.

### Statistical analysis

GraphPad Prism 4 was used for the statistic calculations. The data were not adjusted for sex since the incidence ratio of colorectal cancer between genders (Male: 1128 and female: 1217 new cases in 2004) is 1:1 in Norway [16]. P values < 0.05 were considered significant for all statistical tests.

## Results

### Expression of NDRG2 mRNA in colonic adenomas and carcinomas

Preliminary experiments using a Cancer Profiling Array indicated that expression of *NDRG2 *mRNA was reduced in 9 out of 10 colonic tumors (Table 2). Using a two-tailed paired t-test, the decrease in *NDRG2 *expression in tumors as compared to the corresponding normal tissues was found to be statistically significant (p < 0.01). Prompted by this finding, we decided to analyse *NDRG2 *mRNA expression in normal and neoplastic tissue in a larger number of patients to confirm and extend our results.

**Table 2:** Expression analysis of *NDRG2 *in colon cancer using a Cancer Profiling Array.

**Sample**	**Signal intensity**	**Tumor/normal**	**TNM staging**	**Age**	**Gender**
Tumor/normal	18370/60015	0.31	II; T4N0M0	67	Female
Tumor/normal	31402/35431	0.89	I; T1N0M0	58	Female
Tumor/normal	57125/96121	0.59	IV; T4N0M0	43	Female
Tumor/normal	20382/23650	0.86	IIIB; T3N1M0	69	Female
Tumor/normal	28165/59167	0.48	IIIB; T3N1M0	35	Female
Tumor/normal	22193/12912	1.72	IIIA; T4N0M0	58	Male
Tumor/normal	18569/28122	0.66	IIIB; T4N1M0	63	Male
Tumor/normal	9863/28914	0.34	IIA; T2N0M0	73	Female
Tumor/normal	21780/34774	0.63	IIIB; T4N0M?	65	Female
Tumor/normal	22429/45939	0.49	IIIA; T3N1M0	65	Female

Expression of *NDRG2 *mRNA was determined by hybridisation of a Cancer Profiling Array with a *NDRG2 *probe. Quantification of the hybridisation signal for the paired samples revealed that *NDRG2 *expression was reduced in 9 out of 10 colon tumor samples compared to the corresponding normal sample. Using a two-tailed paired t-test, the decrease in *NDRG2 *expression in tumors compared to normal samples was found to be statistically significant (p < 0.01). The tumors were all diagnosed as adenocarcinoma aside from the Stage I tumor which was a tubulovillous adenoma. The TNM staging system describes the extent of the primary tumor (T), the absence/presence of metastasis to nearby lymph nodes (N) and the absence/presence of distant metastases (M).

**Figure 1:**
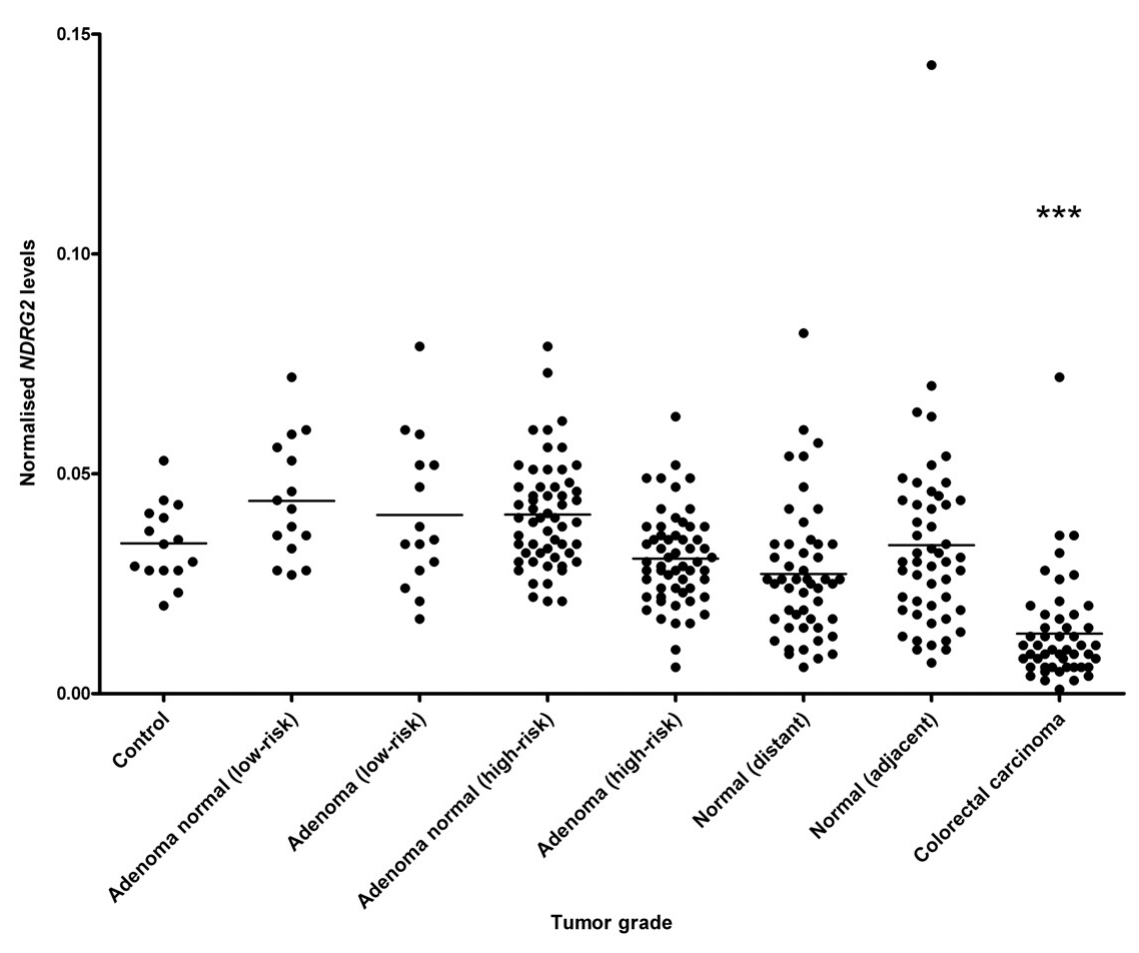
***NDRG2 *mRNA levels are down-regulated during colorectal cancer carcinogenesis**. mRNA expression of *NDRG2 *determined by real-time RT-PCR and normalised to *β-actin *in healthy individuals (Control), normal and affected tissue from the same individual with adenomas (low- or high-risk) and colorectal carcinoma. Normal (adjacent): normal sample close to the carcinoma, Normal (distant): normal sample far from the carcinoma. Each dot represents mean values of triplicate determinations. *** p < 0.001 compared to the control group using one-way ANOVA with a Tukey's post test. A trend of decreased *NDRG2 *expression with increasing tumor grade was observed in affected tissue (p < 0.001).

Using real-time RT-PCR we have measured the levels of *NDRG2 *mRNA in colonic tissue from healthy individuals and from individuals with colorectal adenomas or carcinoma (Table 1). It was observed that the expression of *NDRG2 *mRNA in colorectal tissue is relatively low compared to the expression of *β-actin*, which was used for normalisation (Figure 1). Upon examination of the mean values in affected tissue (low- and high-risk adenomas and carcinoma), a trend towards a decreased *NDRG2 *expression with increasing tumor grade was observed (p < 0.001) (Figure 1).

Analysis of the data using a one-way ANOVA with Tukey's post test did not show any significant difference in *NDRG2 *mRNA level between the control group and either normal or affected tissue from individuals with adenoma (low- and high-risk). This was also the case when comparing normal tissue from individuals with colorectal cancer to the control group. However, when comparing affected tissue from individuals with colorectal cancer to corresponding tissue from a healthy control, a statistically significant difference in the level of *NDRG2 *mRNA was observed (p < 0.001) (Table 3).

**Table 3:** Mean values of normalised levels of *NDRG2 *mRNA in normal and affected colonic tissues.

	**mRNA level in normal tissue Mean **(SD)	**p^a^**	**mRNA level in adenomas/carcinomas Mean **(SD)	**p^a^**	**p^b^**
**Control**	**0.034 **(0.009)				
**Low-risk Adenoma**	**0.044 **(0.014)	**NS**	**0.0407 **(0.017)	**NS**	**NS**
**High-risk Adenoma**	**0.041 **(0.012)	**NS**	**0.0307 **(0.011)	**NS**	**< 0.001**
**Carcinoma Normal (distant)**	**0.027 **(0.015)	**NS**	**0.0136 **(0.012)	**< 0.001**	**< 0.001**^c^
**Carcinoma Normal (adjacent)**	**0.034 **(0.021)	**NS**			**< 0.001**^d^

NS = not significant.

^a ^p value when expression levels were compared to the control group of healthy individuals using one-way ANOVA with a Tukey's post test.

^b ^p value for comparison of mRNA expression levels in normal and tumor samples from the same individual using a paired two-tailed T-test.

^c ^p value when normal (distant) and corresponding tumor sample were compared.

^d ^p value when normal (adjacent) and corresponding tumor sample were compared.

Further analysis of the different groups of affected tissue using a paired two-tailed t-test showed that the level of *NDRG2 *in individuals with low-risk adenoma did not show any significant difference between normal and neoplastic tissue. However, a comparison of normal and high-risk adenoma from the same individual showed a highly statistically significant reduction (p < 0.001) in *NDRG2 *level. Finally, comparing the level of *NDRG2 *mRNA in normal tissue far (normal distant) and close (normal adjacent) to that of the tumor in the surgical specimens of CRC patients showed a statistically significant difference (p < 0.001) in both cases (Table 3).

Cases of adenomas can also be classified according to the diagnosed degree of dysplasia (mild/moderate or severe) (Table 4). When comparing affected tissue with normal tissue from the same individual, we found a statistically significant difference in *NDRG2 *expression for individuals with mild/moderate dysplasia (p < 0.001) (Table 4).

**Table 4:** Mean values of normalised levels of *NDRG2 *mRNA in adenomas^1^.

	**mRNA level in normal tissue Mean **(SD)	**p^a^**	**mRNA level in adenomas Mean **(SD)	**p^a^**	**p^b^**
**Mild/moderate Dysplasia**	**0.042 **(0.012)	**NS**	**0.032 **(0.014)	**NS**	**< 0.001**
**Severe Dysplasia**	**0.040 **(0.014)	**NS**	**0.034 (**0.011)	**NS**	**NS**

NS = not significant.

^1^Cases of adenomas were divided into mild/moderate (n = 52) or severe (n = 20) according to the diagnosed degree of dysplasia.

^a ^p value when expression levels were compared to the control group of healthy individuals using one-way ANOVA with a Tukey's post test.

^b ^p value for comparison of mRNA expression levels in normal and tumor samples from the same individual using a paired two-tailed T-test.

### Analysis of expression levels in carcinomas according to Dukes' staging

**Figure 2:**
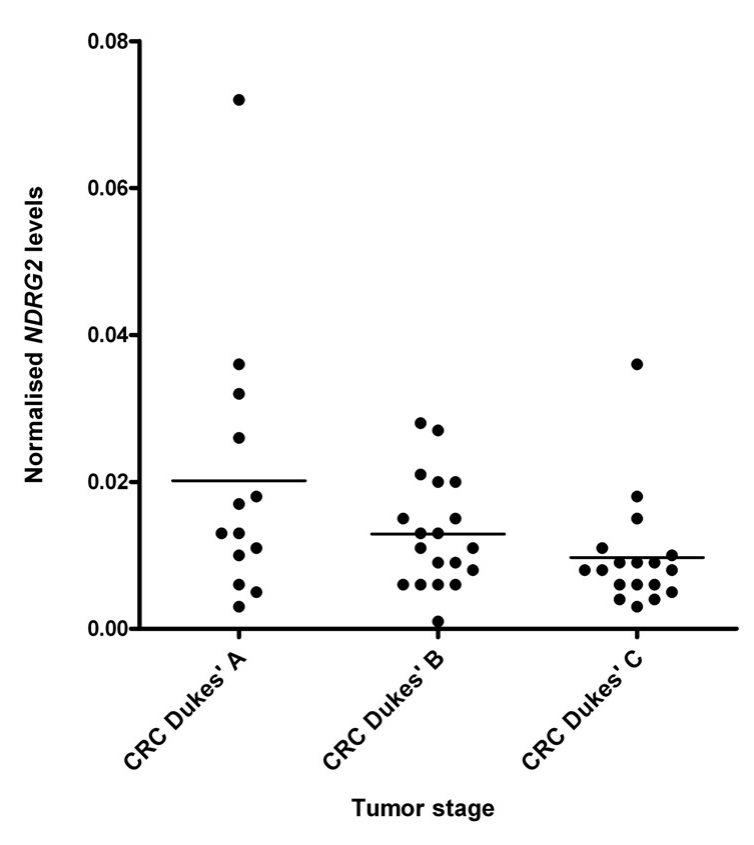
**NDRG2 mRNA levels decrease with increasing Dukes' stage**. Samples with colorectal cancer (CRC) staged after the Dukes' staging system with 13 samples categorised as Dukes' A, 19 samples as Dukes' B and 18 samples as Dukes' C. The graph shows the normalised level of *NDRG2 *mRNA in samples from the different Dukes' stages. Calculating linear regression using each column of data resulted in a statistically significant linear trend (p < 0.05) for a decrease in *NDRG2 *level with increasing Dukes' stage.

Colorectal cancer can be staged according to different systems. In this study all samples of colorectal carcinoma (CRC) were classified as Dukes' stage A-C where C is the most advanced and metastatic stage. Figure 2 presents data showing that the level of *NDRG2 *mRNA decreases with increasing Dukes' stage. By calculating linear regression on the data, the result was a statistically significant linear trend (p < 0.05) for decreasing *NDRG2 *levels with increasing tumor stage.

### Expression patterns of NDRG2 between genders in colorectal cancer

**Figure 3:**
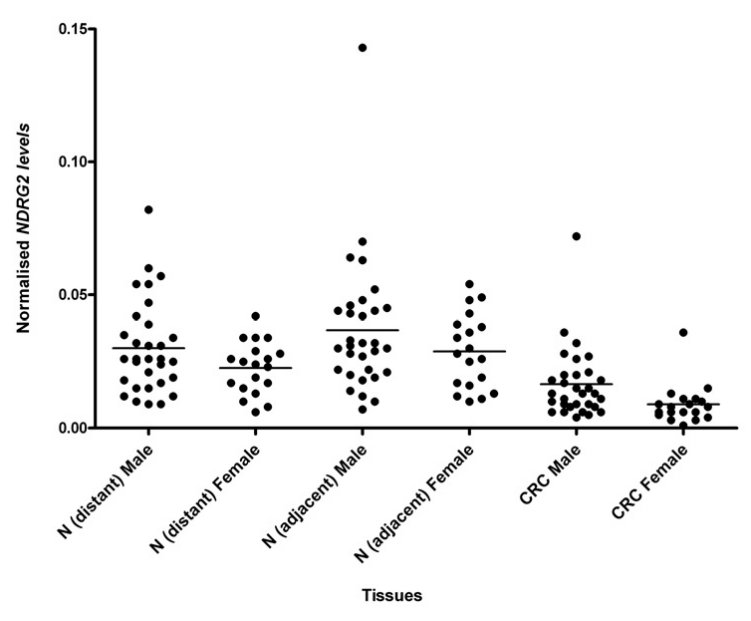
**NDRG2mRNA levels are lower in females compared to males**. Expression levels of *NDRG2 *mRNA in samples with colorectal carcinomas (CRC) divided according to gender. A general lower level of expression was observed in females. N (adjacent): normal sample close to the tumor, N (distant): normal sample far from the tumor.

The incidence of new cases of colorectal cancer in Norway is in the ratio 1:1 between the two genders [16]. Dividing all data collected in this study into groups of males and females showed a general lower level of *NDRG2 *expression in females with colorectal carcinoma (both normal and cancer tissue) (Figure 3). However, this difference was not statistically significant using a one-way ANOVA with Tukey's post test.

## Discussion

In the present study we demonstrated that *NDRG2 *mRNA expression levels were lower in colonic tumors than in normal colon tissue from the same individual. This was observed using two distinct subject populations, one of which was a Norwegian cohort, the other a group of affected individuals based on a commercially available product. The difference in mRNA level is likely to be reflected at the level of NDRG2 protein, since *NDRG2 *mRNA levels have previously been shown to correlate well with protein levels [8].

In the Norwegian cohort, *NDRG2 *mRNA levels were statistically significantly reduced in colorectal carcinoma when compared to the healthy controls. In order to examine whether the risk of carcinoma is affected by changes in the microenvironment, expression levels of *NDRG2 *in the lesion were compared to normal adjacent tissue as well as to normal tissue distant from the tumor. *NDRG2 *mRNA was statistically significantly reduced in tumor compared to either normal tissue sample. No difference was observed between the adjacent and distant samples, suggesting that changes in *NDRG2 *expression in the carcinoma are not attributable to the microenvironment.

Recent studies have demonstrated that colorectal cancer is a heterogenous disease with distinct molecular components. Distinct genetic or epigenetic alterations have been identified which correlate with the location of the tumors [17]. Although it was not investigated in this study, it could be interesting to compare *NDRG2 *expression in tumors located in either the proximal or distal colon.

There was a tendency for decreasing *NDRG2 *mRNA levels with increasing tumor stage according to Dukes' staging of the CRC samples, and this trend was found to be significant using linear regression (p < 0.05). Our results are in agreement with that observed for other cancer types where *NDRG2 *expression is reduced in high-grade compared to low-grade tumors [4,10]. This trend indicates either that the loss of *NDRG2 *promotes tumor progression or that *NDRG2 *is inactivated by factor(s) present at advanced tumor stages. *NDRG2 *has previously been shown to be negatively regulated by the c-Myc oncoprotein [8] and it is possible that elevated levels of c-Myc would result in reduced expression of *NDRG2*. Thus, it could be interesting to elucidate whether or not an increased level of c-Myc, which is a frequent event in colorectal cancer [18], correlates with a decreased level of *NDRG2*. Measurement of c-Myc levels was not included in these studies. However, we have investigated a subset of the CRC samples (n = 54) from the KAM study for β-catenin expression by immunohistochemistry. All of the tested CRC samples are positive for cytoplasmic β-catenin and 72% are β-catenin positive in all nuclei (data not shown). The remaining samples contain nuclear β-catenin in occasional nuclei. This suggests that c-Myc levels are likely to be elevated since c-Myc is known to be positively regulated by nuclear β-catenin [19].

In order to determine the stage at which *NDRG2 *expression is down-regulated in the adenoma-carcinoma sequence we also examined normal and affected tissue from low- and high-risk adenomas. When comparing affected tissue with normal tissue from the same individual, we found a statistically significant difference for individuals with high-risk adenomas. However, when compared to the control group of healthy individuals, only the affected tissue from individuals with colorectal carcinoma shows a statistically significant reduction in *NDRG2 *mRNA levels. Our results suggest that down-regulation of *NDRG2 *expression occurs during the progression from adenoma to carcinoma.

Whether down-regulation of *NDRG2 *in colorectal carcinoma is a cause or a consequence of malignant progression is at present unclear. Although the structure of NDRG2 resembles that of a hydrolase [2], its ability to function as an enzyme is presently unknown. It has recently been shown that overexpression of *NDRG2 *in a glioblastoma cell-line inhibits cell proliferation [4] and that *NDRG2 *expression correlates positively with survival in high-grade glioma [12]. *NDRG2 *levels are also reduced in several cancer types and cell-lines [4,9-11]. Thus, *NDRG2 *may have a general function in diverse tissues as a tumor suppressor gene. Future studies will be needed to examine whether increased *NDRG2 *levels in colorectal carcinoma correlate with improved prognosis.

## Conclusion

In conclusion, *NDRG2 *mRNA levels were decreased in both high-risk colorectal adenoma and in colorectal carcinoma compared to corresponding normal colonic mucosa from the same individual. Furthermore, *NDRG2 *expression was reduced in colorectal carcinoma compared to normal tissue from healthy individuals. Our results suggest that *NDRG2 *down-regulation correlates with the progression of dysplastic tissue to carcinoma. Future studies are needed to address whether *NDRG2 *down-regulation is a cause or consequence of colorectal carcinogenesis.

## Competing interests

The author(s) declare that they have no competing interests.

## Authors' contributions

LKV and CM conceived the idea of the study and designed the primers and probes. EHK designed and administered the KAM study and collected the samples. LKV, MS and CFS extracted the RNA and carried out the cDNA synthesis. SG organised the cDNA bank used in this study. GH together with KMT were responsible for designing and administering the NORCCAP clinical trial. IMBL was responsible for the pathology of the cancer cases. TI contributed with scientific input to the study. AL validated the primers and probes, carried out the RT-PCR and performed most of the statistical calculations. RL carried out the Cancer Profiling Array analysis. CM and AL drafted the manuscript. LKV helped writing the manuscript. All authors contributed to interpretation and discussion of the results and read and approved the final version.

## Pre-publication history

The pre-publication history for this paper can be accessed here:


